# Effect of bladder distension on arginine vasopressin secretion in rats

**DOI:** 10.1186/s13104-019-4105-7

**Published:** 2019-02-02

**Authors:** Yosuke Morizawa, Kazumasa Torimoto, Shunta Hori, Daisuke Gotoh, Yasushi Nakai, Makito Miyake, Kiyohide Fujimoto

**Affiliations:** Department of Urology, Nara Medical University, 840 Shijo-cho, Kashihara, Nara 634-8522 Japan

**Keywords:** Bladder distention, AVP, Rat

## Abstract

**Objective:**

Urine production is regulated throughout the night to ensure that it remains within functional bladder capacity. Increasing bladder capacity may thus play an important role in urine production. We investigated arginine vasopressin (AVP) secretion in female rats anesthetized with urethane in the daytime and nighttime under distended/empty bladder conditions. Chronological serum AVP was measured by enzyme immunoassay in rats with distended or empty bladders.

**Results:**

The mean baseline AVP level was significantly higher in the nighttime than in the daytime (17.21 ± 5.29 vs 11.68 ± 3.16 pg/mL, p = 0.0019). However, serum AVP levels did not change with time in the daytime or nighttime, regardless of an empty or distended bladder. We therefore concluded that AVP secretion was unaffected by bladder filling.

## Introduction

Arginine vasopressin (AVP) is a neurohypophyseal hormone released primarily in response to increasing plasma osmolarity in the absence of significant hypovolemia. Intracorporeal fluid retention, urine production, and urine osmolarity are regulated by circadian changes in AVP secretion [1]. Various antidiuretic mechanisms, including decreased renal blood flow associated with the recumbent position and increased secretion of AVP, are responsible for reducing urine production by the kidneys during sleep. The urinary bladder generally expands with continuing urine production during sleep, until the individual is awakened by the need to void when the bladder becomes full. However, although the bladder stores urine up to its functional capacity at night, it does not continue to expand during the day [2]. These results suggest that urine production is regulated throughout the night to ensure that it does not exceed the functional bladder capacity, and night-time urine production may thus be regulated by increasing the bladder capacity, such as by bladder distension. A reduced bladder capacity and the lack of a normal diurnal AVP rhythm, leading to increased night-time AVP secretion, are important etiologies of nocturia [3–5]. These observations suggest that a reduced bladder capacity and the secretion of AVP may be closely related. We therefore investigated changes in AVP secretion under different bladder conditions in rats.

## Main text

### Materials and methods

#### Animals

Female Sprague–Dawley rats weighing 300 g were obtained from Oriental Bio Service (Kyoto, Japan) and housed in groups of two per cage under a 12-h light/12-h dark cycle (lights on automatically at 08:00) with access to food and water ad libitum. Animal care was carried out in compliance with the recommendations of The Guide for Care and Use of Laboratory Animals (National Research Council) and the study was approved by the animal facility committee at Nara Medical University.

#### Arginine vasopressin measurements

Thirty-six rats were divided into six groups (n = 6 each) according to experiment time and bladder condition: control daytime; control nighttime; empty bladder daytime; distended bladder daytime; empty bladder nighttime; and distended bladder nighttime. Experiments in the daytime groups were performed between 09:00 and 11:00, and experiments in the nighttime groups between 21:00 and 23:00. The rats were anesthetized with urethane (1.0 g/kg body weight, intraperitoneal injection) and ureters in rats the empty- and distended-bladder groups were ligated bilaterally at the level of bifurcation of the abdominal aorta. A transurethral bladder catheter (PE50) was inserted into the proximal urethra, which was ligated to prevent leakage of intravesical fluid. The bladder was left empty in the empty-bladder group and was filled with 1.0 mL of saline in the distended-bladder group. No ureter ligation or transurethral catheterization was performed in the control groups. AVP has a very short half-life of 16–24 min, and blood samples were therefore collected from the internal jugular vein at 0, 30, and 60 min after the procedure. Serum AVP was measured using a Vasopressin Enzyme Immunoassay Kit (RayBiotech, GA, USA).

#### Statistical analysis

Baseline AVP levels between the daytime and nighttime groups were compared with Mann–Whitney test. Baseline serum AVP levels and chronological changes in serum AVP were compared with Friedman test. Data were analyzed and plotted using PRISM software version 7.04 (GraphPad Software, Inc., San Diego, CA, USA). All the data were expressed as mean ± standard deviation. A p-value < 0.05 was defined as statistically significant.

### Results

**Fig. 1:**
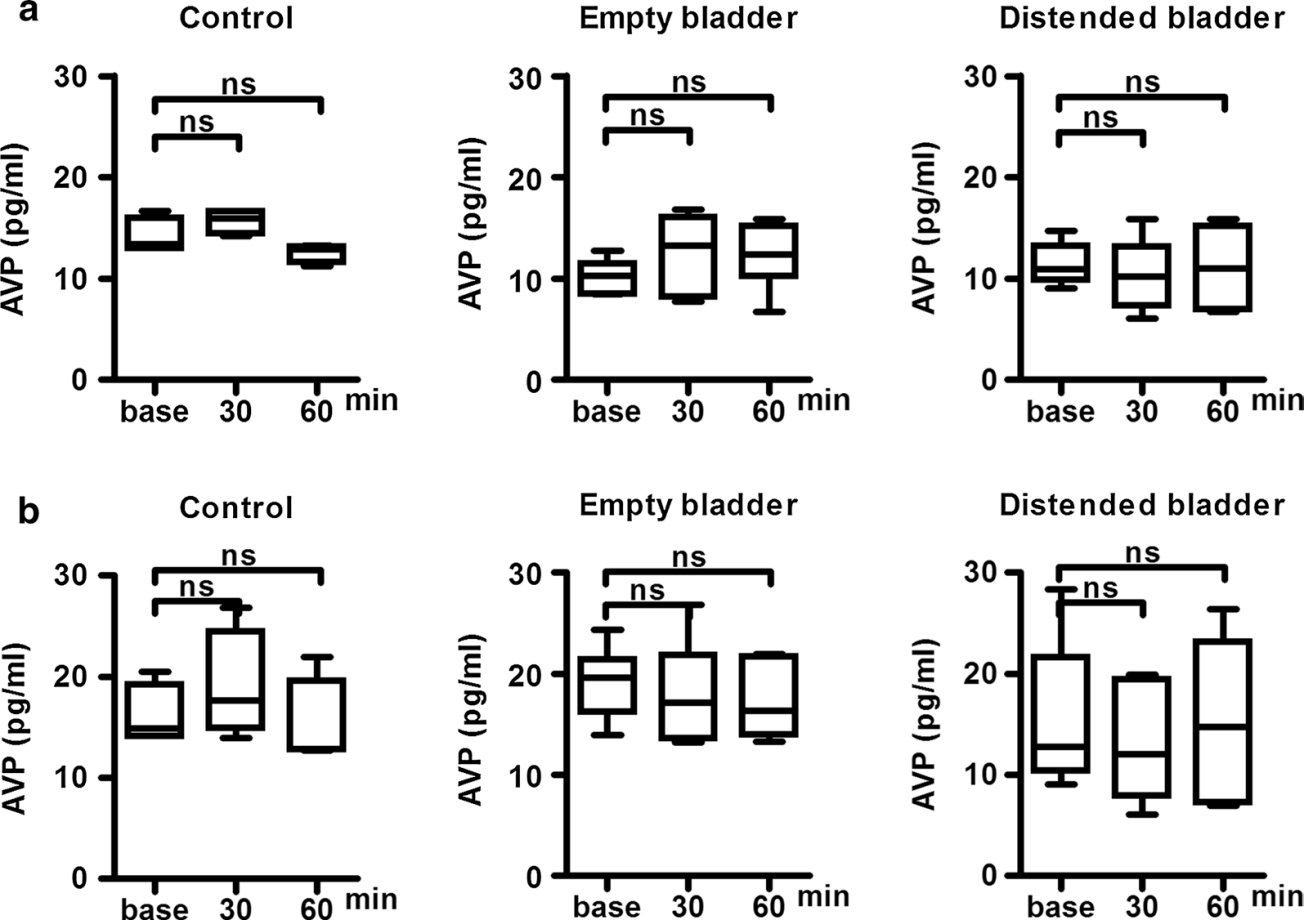
Chronological serum AVP changes in control, empty bladder and distended bladder group rats. **a** Chronological changes in serum arginine vasopressin (AVP) in the daytime groups. Rats were divided into three (n = 6 each): Control; empty bladder; and distended bladder. Experiments were performed between 9:00 and 11:00. Serum AVP was measured at 0, 30, and 60 min after the transurethral catheterization or bladder distention. The AVP levels did not change from baseline in each three groups. **b** Chronological changes in serum AVP in the nighttime groups. Rats were divided into three groups (n = 6 each): control; empty bladder; and distended bladder. Experiments were performed between 21:00 and 23:00. Serum AVP was measured at 0, 30, and 60 min after the transurethral catheterization or bladder distention. The AVP levels did not change from baseline in each three groups

The mean baseline AVP level was significantly higher in the nighttime groups than in the daytime groups (17.21 ± 5.29 vs 11.68 ± 3.16 pg/mL, p = 0.0019). AVP levels remained unchanged up to 60 min in the control groups (n = 6) without ureter ligation and transurethral catheterization, indicating that AVP secretion was unaffected by surgical stress such as transurethral catheterization. AVP levels also remained unchanged up to 60 min in the daytime (Fig. 1a) and nighttime groups, regardless of an empty or distended bladder (Fig. 1b). AVP secretion was therefore unaffected by bladder distension.

### Discussion

To the best of our knowledge, this is the first study to report on the effects of a distended bladder condition on AVP secretion in rats. Kawauchi et al. reported that patients who underwent total cystectomy and ileal conduit lacked a circadian AVP rhythm [6], while Szollar et al. also reported the absence of a circadian AVP rhythm in spinal cord injury patients with urinary catheterization for bladder management [7]. Clinical studies thus suggest that the lack of bladder distention may induce the absence of a circadian AVP rhythm. The bladder may regulate AVP secretion and may indirectly affect water metabolism, in addition to the kidney. However, in contrast, the results of the present study in rats indicated that AVP secretion was unaffected by bladder distension. In previous studies, the AVP concentration readily increased in response to various conditions, such as manual contact and anesthesia [8, 9]. In the present study, the AVP concentration was higher than in a previous study involving a blood sampling technique for measurement of AVP [10]. Surgical stress, urethral catheterization, multiple blood samplings, and anesthesia might influence the AVP concentration. Therefore, the change in the AVP concentration in the distended bladder condition might be undetectable.

Anticholinergic agents increase the bladder capacity by acting on bladder C-fibers and thus decrease nocturnal urine volume in patients with nocturnal polyuria [11]. Another rat study reported that imidafenacin exerted antidiuretic effects via the AVP signaling pathway [12]. Increasing bladder capacity by antidiuretic agents may thus affect the AVP signaling pathway. Bladder capacity in children has been shown to be > 30% larger than functional bladder capacity in the morning, immediately after awakening [13]. Increasing bladder capacity at night may regulate urine production via the AVP signaling pathway. We hypothesized that increasing bladder capacity, such as in the distended bladder in night, might regulate urine production by enhancing the AVP signaling pathway. However, AVP secretion in the current rat model was unaffected by a change in bladder condition, suggesting that bladder distension was not equivalent to increasing bladder capacity. Further studies are needed to elucidate the detailed mechanisms of urine production in relation to AVP secretion and bladder capacity.

## Limitations

The goal of this study was to estimate the elevation of AVP due to bladder distention in rat study. However, we did not measure the direct change of AVP in different bladder condition. We hypothesize that urine production in night may be regulated by the change of bladder capacity. Probably increasing bladder capacity in night may not regulate urine production by the AVP signaling pathway but by another mechanism. We need to perform further well-designed studies to confirm the association urine production and bladder capacity.

## Abbreviation

AVP: arginine vasopressin
